# MiR-129-2 functions as a tumor suppressor in glioma cells by targeting HMGB1 and is down-regulated by DNA methylation

**DOI:** 10.1007/s11010-015-2382-6

**Published:** 2015-03-14

**Authors:** Yu Yang, Jun-Qiang Huang, Xi Zhang, Liang-Fang Shen

**Affiliations:** 1 Department of Oncology, Xiangya Hospital, Central South University, Changsha, 410008 Hunan China; 2 Department of Oncology, 163 Hospital of PLA, Changsha, 410003 China; 3 Department of Neurosurgery, The First Hospital of Changsha, Changsha, 430100 China; 4 Department of Oncology, The Third Xiangya Hospital, Central South University, Changsha, 430100 China

**Keywords:** Glioma, miR-129-2, High-mobility group box 1, DNA methylation

## Abstract

MicroRNA (miRNA) dysregulation is causally related to cancer development and progression, and recent reports have revealed that DNA methylation constitutes an important mechanism for miRNA deregulation in cancer. MiR-129-2 has been reported to be down-regulated and functions as a tumor suppressor in a few human cancers. However, the involvement of miR-129-2 in the pathology of glioma and the mechanism underlying miR-129-2 regulation in glioma cells remain unclear. In this study, we performed quantitative PCR to investigate the level of miR-129-2 in 21 pairs of glioma tumors and matched adjacent tissues and found that miR-129-2 is down-regulated in glioma tumors. In vitro cell growth, apoptosis, cell migration, and invasion assays revealed that miR-129-2 functions as a tumor suppressor in glioma cells. Luciferase reporter assay found that miR-129-2 could directly target high-mobility group box 1 (HMGB1) and inhibit its expression in glioma cells. Methylation-specific PCR found that DNA methylation in upstream regions of miR-129-2 occured more frequently in cancer tissues than in adjacent tissues. Demethylation of miR-129-2 by 5-aza-2′-deoxycytidine treatment and quantitative PCR analysis revealed that miR-129-2 expression is epigenetically regulated in glioma cells. Taken together, our data suggested that miR-129-2 functions as a tumor suppressor in glioma cells by directly targeting HMGB1 and is down-regulated by DNA methylation, which may provide a novel therapeutic strategy for treatment of glioma.

## Introduction

Glioma is the majority of malignant brain tumors and represents a serious health problem worldwide [1]. Although glioma has been widely studied, the molecular mechanisms underlying its pathology remain poorly understood.

MicroRNAs (miRNAs) are a group of endogenous small noncoding RNAs that are involved in the posttranscriptional regulation of gene expression through imperfect base-pairing with the 3′untranslated region (3′UTR) of target mRNAs [2, 3]. MiRNAs have been found to be critical in numerous biological functions such as cell proliferation, differentiation, and apoptosis [4]. Accumulated evidence has also revealed that miRNAs are deregulated in various diseases, including human cancer [5–7].

MiR-129 family members have been reported to function as a tumor suppressor in a variety of human cancers. For example, miR-129 was down-regulated in gastric cancer and increased miR-129 in gastric cancer cells resulted in significant G0/G1 phase arrest [8]. In esophageal carcinoma cells, miR-129-2 suppresses cell proliferation and migration through down-regulation of SOX4 expression [9]. However, the function of miR-129 and the mechanism underlying glioma carcinogenesis remain unclear. In this study, we examined the expression of miR-129-2 in glioma tumors and cell lines and investigated the roles of miR-129-2 in glioma cells. We found that miR-129-2 was down-regulated in glioma tumors and cell lines, while enforced expression of miR-129-2 repressed glioma cell growth, cell migration, and invasion and promoted cell apoptosis in vitro. Luciferase reporter assay further revealed that miR-129-2 could directly target and inhibit high-mobility group box 1 (HMGB1), which has been found to be up-regulated and involved in the pathogenesis of many kinds of human cancers [10], and knockdown of HMGB1 inhibits cell migration and invasion of glioma cells in vitro in our study.

Epigenetic modifications are closely associated with gene expression. For example, promoter hypermethylation is thought to be a mechanism to down-regulate tumor suppressor genes in human cancers [11]. Recently, miRNAs whose expression is repressed by DNA methylation have been reported in a few human cancers [12–14]. As for miR-129-2, it has been reported to be frequently methylated in hepatocellular carcinoma and gastric cancer [15–17], and methylation-mediated repression of miR-129-2 enhanced oncogenic SOX4 expression in HCC [18], gastric cancer [19], and endometrial cancer [20]. In this study, to investigate whether the down-regulation of miR-129-2 originates from the hypermethylation of the miR-129-2 genomic region, we analyzed the DNA methylation of CpG island in the upstream region of miR-129-2 in glioma tumors and found that down-regulation of miR-129-2 in glioma tumors might be due to the hypermethylation of CpG sequences in its upstream region.

## Materials and methods

### Tissue samples and cell lines

Clinical tumor tissues and the corresponding adjacent tissues were collected from 21 glioma patients from Xiangya Hospital and were stored at −80 °C until RNA extraction. Four human glioma cell lines including A172, U251, U373, U87, and a normal neuronal cell line primary human fetal glial (PHFG cell) were preserved in our laboratory and maintained in DMEM or RPMI 1640 with 10 % fetal bovine serum (FBS) in a humidified atmosphere of 5 % CO_2_ at 37 °C. The Clinical Research Ethics Committee of Central South University approved the research protocols, and written informed consent was obtained from the participants.

### RNA extraction and reverse-transcribed quantitative PCR (RTq-PCR)

Total RNA was extracted from tissues and cell lines using Trizol reagent (Invitrogen, CA, USA) according to the manufacturer’s instructions. 1 μg of total RNA was reversely transcribed using oligo (dT) 18 as the RT primers for reverse transcription of mRNA and a stem-loop RT primer for the reverse transcription of miRNA. Quantitative PCR was then performed using ABI 7500 Sequence Detection System (Life Technologies, NY, USA). For mRNAs, the data were normalized using the endogenous β-actin as control. The specific primers for β-actin and HMGB1 are as follows: β-actin-F: 5′-AGGGGCCGGACTCGTCATACT-3′; β-actin-R: 5′-GGCGGCACCACCATGTACCCT-3′; HMGB1-F: 5′-AGAAGTGCTCAGAGAGGTGGA-3′; HMGB1-R: 5′-CCTTTGGGAGGGATATAGGTT-3′. To quantify the miR-129-2 expression levels, the expression of small nuclear U6 was used as internal control. The specific primers for miRNA-129b and U6 were purchased from GeneCopoeia. All experiments were performed in triplicate. Relative expression levels were calculated using the 2^−∆∆*Ct*^ method.

### MTT assay

Cells were allowed to grow in 96-well plates with 5000 cell per well and incubated for 24, 48, and 72 h and then MTT (10 mg/ml) was added to the cells and incubated for 3 h. The reaction was then terminated by removal of the supernatant followed by adding 200 μl of DMSO. After 2-h incubation, the optical density at 570 nm of each well was measured with a microplate reader (Bio–Rad).

### Cell migration and invasion assays

Cell migration was assessed by wound-healing assay. An artificial wound was scratched on a confluent cell monolayer without FBS using sterile tips, and wound-healing images were taken at 24 and 48 h later. Cell invasion was assessed using transwell invasion chambers coated with matrigel (BD Biosciences, Franklin Lakes, NJ, USA). 0.2 ml of cells suspended in serum-free medium was added into the upper chamber. The lower chamber was filled with 500 ul of RPMI 1640 or DMEM medium with 10 % FBS as the nutritional attractant. 24 h later, cells remaining on the upper side of the membrane were removed, and cells that migrated through the membrane were fixed with 75 % alcohol and stained with crystal violet, and the invasive cells were counted and imaged using an inverted microscope (Nikon, Japan).

### Cell cycle and apoptosis by flow cytometric analysis

Cell cycle analysis was performed by flow cytometric (FCM) analysis. The cells were fixed in 70 % ethanol, washed with PBS, and resuspended in staining solution (50 μg/ml of propidium iodide, 1 mg/ml of RNase A, 0.1 % Triton X-100 in PBS). After incubation for 30 min at 4 °C, the stained cells were then analyzed with a flow cytometer (Beckman Coulter). For apoptosis assay, cells were collected and transferred to 60 mm dishes. The cell apoptosis ratio was analyzed using the Annexin V-FITC Apoptosis Detection kit (BD Biosciences, San Diego, CA), according to the manufacturer’s instructions.

### Western blotting

Total cellular extracts were prepared with lysis buffer, and approximately 50 μg of total protein was separated by SDS-PAGE, transferred to a PVDF membrane, and incubated with the antibodies, followed by the HRP-conjugated secondary antibody. Signals were visualized using ECL substrates (Millipore, USA). The protein bands were visualized using the enhanced chemiluminescence (ECL) detection kit (Amersham) as recommended by the manufacturer. β-Actin was used for normalization. Antibodies of HMGB1 and β-actin were obtained from Abzoom (Abzoom, USA).

### Luciferase reporter assays

The 3′UTR of the wild-type HMGB1 and a variant containing mutations in the putative miR-129-2 binding sites (Fig. 3a) were inserted downstream of the firefly luciferase reporter in the psiCHECK-2 vector (Promega, Madison, WI, USA). U373 and U87 cells were seeded into 24-well plates for 24 h before transfection. Cells were then co-transfected with the reporter vector (psiCHECK-2-HMGB1-WT-3′UTR or psiCHECK-2-HMGB1-Wut-3′UTR) and miR-129-2 mimics or scrambled mimics using Lipofectamine 2000 (Invitrogen, Carlsbad, CA, USA). Cells were harvested, and luciferase activity was detected using a dual-luciferase reporter assay system (Promega, Fitchburg, WI, USA) 48 h after transfection. All experiments were performed in triplicate. The miR-129-2 mimics, miR-129-2 inhibitor, and their scrambled mimics (negative control) were purchased from Genechem (Shanghai, China).

### Lentivirus infection and siRNAs

Lentiviruses containing miR-129-2 (Lv-miR-129) and negative control (Lv-NC) were purchased from GeneChem (Shanghai, China). Cells were cultured up to 70 % of the plates and then added by a concentration of 2.4 × 10^5^ TU/well Lv-miR-129-2 or negative control lentivirus. Quantitative PCR was then performed to determinate the expression levels of miR-129-2 and HMGB1 after being infected for 6 days. The small interfering RNAs (siRNA) targeting human HMGB1 mRNA and negative control siRNA (si-NC) were purchased from Ruibobio (Guangzhou, China).

### 5-Aza-dC treatment

For demethylation study, U373 and U87 cells were treated with 5-aza-2′-deoxycytidine (5-Aza-dC, Sigma-Aldrich, USA) at 0.5 mmol/l for 4 days. Culture medium containing 5-Aza-dC was replaced every 24 h.

### DNA bisulfite modification and bisulfite-sequencing PCR

Genomic DNA was obtained from tissues or cells using DNA Extraction Mini Kit (TIANGEN Biotech, Beijing, China) according to the manufacturer's instructions. 1 μg of genomic DNA was bisulfite treated and recovered with EZ DNA Methylation-Gold kit (Zymo Research, CA, USA) according to the manufacturer’s instructions. Bisulfite-sequencing PCR (BSP) for miR-129-2 was done using ZymoTaqTM PreMix (Zymo Research) with the following primers: 5′-TGATAGGGAGATAGAGGGAT-3′ (forward) and 5′-AACAAACTAAATCTCCCCAA-3′ (reverse), which amplify a 268-bp product under the conditions of 95 °C for 10 min, followed by 40 cycles of 95 °C for 30 s, 58 °C for 30 s, and 72 °C for 30 s. The BSP products contained 22 CpG sites in the promoter region of miR-129-2 and were cloned into the pUC18-T vector (Biodee, Beijing, China). Twenty clones for each group were randomly selected and sequenced by Shanghai Sangon Co (Shanghai, China).

### Methylation-specific PCR (MSP)

Methylation-specific PCR (MSP) primers detecting methylated (M) or unmethylated (U) alleles of the miR-129-2 promoter were miR-129-2-MF, 5′-TTTTAGTTCGTATTAATGAGTTGGC-3′ and miR-129-2-MR, 5′-AATCTCTAAACAAATACAATTCGAA-3′ for methylated alleles and miR-129-2-UF, 5′-TTAGTTTGTATTAATGAGTTGGTGG-3′ and miR-129-2-UR, 5′-AATCTCTAAACAAATACAATTCAAA-3′ for unmethylated alleles. MSP was performed for 35 cycles (95 °C for 30 s, 58 °C for 30 s, and 72 °C for 30 s). MSP primers were first checked for not amplifying any unbisulfited DNA. PCR products were then analyzed on a 2 % agarose gel. Samples with a stronger band intensity than the negative control in the MSP were regarded as methylated, and samples with no visible PCR product were regarded as unmethylated.

## Results

### MiR-129-2 was down-regulated in glioma tumors and cell lines

**Fig. 1:**
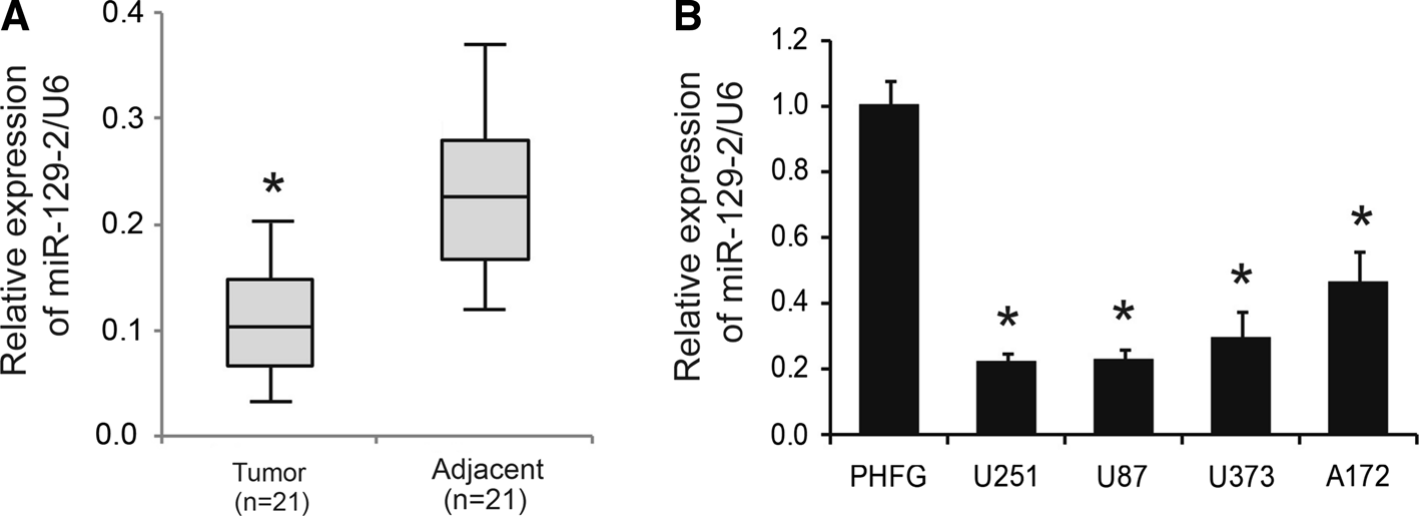
MiR-129-2 was down-regulated in glioma tumors and cell lines. **a** Boxplots to show the relative expression levels of miR-129-2 in 21 pairs of glioma tissues and their matched adjacent tissues measured by RTq-PCR. **b** The relative expression levels of miR-129-2 in normal neuronal cell line PHFG and four glioma cell lines (A172, U251, U373, and U87). U6 was used as an internal control. *P < 0.05 versus the normal

To assess the expression of miR-129-2 in glioma cells, RTq-PCR analysis was performed in 21 pairs of glioma tissues and matched adjacent tissues. As shown in Fig. 1a, the expression levels of miR-129-2 were significantly down-regulated in glioma tumors compared with those in matched adjacent tissues. Additionally, miR-129-2 expression levels in four glioma cell lines (A172, U251, U373, and U87) were also decreased relative to the normal neuronal cell line PHFG (Fig. 1b). Taken together, these data suggest a potential link between loss of miR-129-2 and glioma pathology.

### MiR-129-2 inhibits glioma cell growth and promotes cell apoptosis in vitro

**Fig. 2:**
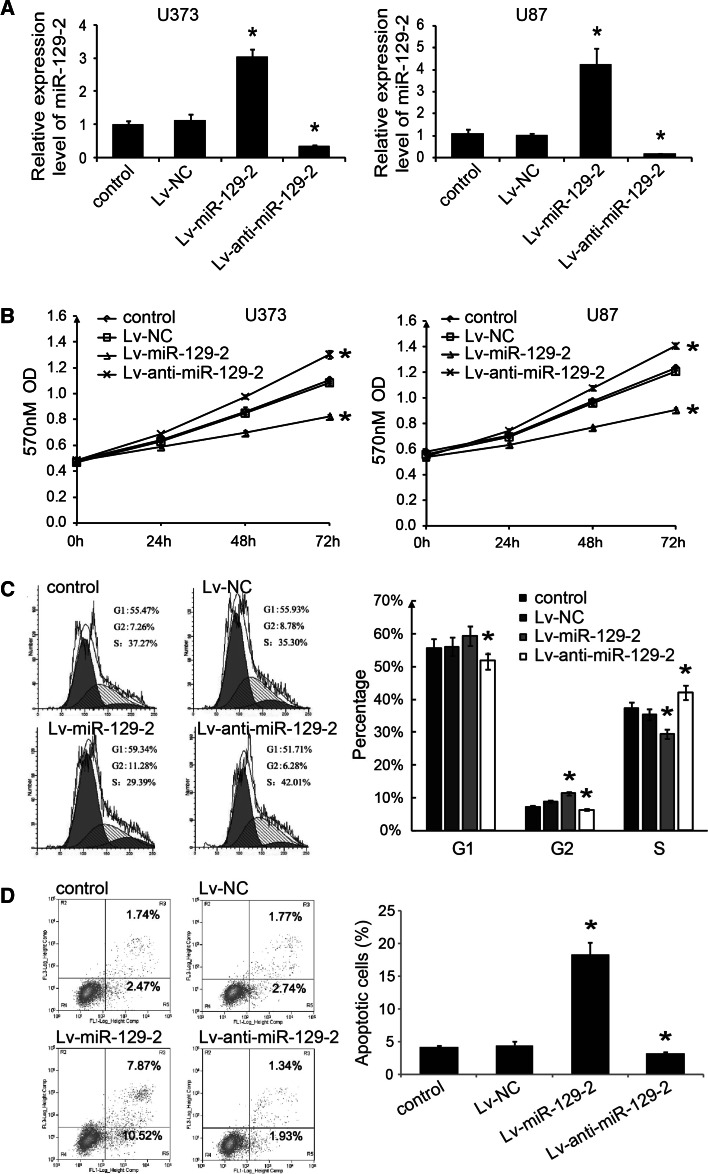
MiR-129-2 inhibits glioma cell growth and promotes cell apoptosis. After lentivirus infection, the expression of miR-129-2 (**a**), the cell proliferation (**b**), the percentage of cells in the G1, G2, and S phases (**c**), and apoptotic cell fractions (both early and late apoptotic cells) (**d**) were determined by RTqPCR, MTT, and FCM analysis, respectively. *P < 0.05 versus the control, data shown are mean ± SD **c** and **d** are for U373 cells, and the similar results were also found in U87 cells (data not shown)

We then investigated the functional roles of miR-129-2 in glioma cells. We constructed lentiviral vector expressing pre-miR-129-2 (Lv-miR-129-2) or miR-129-2 antisense (Lv-anti-miR-129-2) and infected U373 and U87 cells to restore or inhibit miR-129-2 expression. RTq-PCR was performed to confirm that miR-129-2 was restored in these stable infected cells (Fig. 2a). We then investigated the effect of miR-129-2 on cell proliferation, cell cycle, and apoptosis, respectively. MTT assays indicated that the enhanced expression of miR-129-2 could significantly inhibit cell proliferation compared to control group in U373 and U87 cells (Fig. 2b). In addition, we found that miR-129-2 restoration increased the percentages of G1 (not significantly) and G2 phase cells in U373 and U87 cells (Fig. 2c) and promoted cell apoptosis (Fig. 2d), as determined by FCM analysis. In contrast, when endogenous miR-129-2 was silenced, the cell proliferation (Fig. 2b) and cell cycle (Fig. 2c) were significantly increased, and cell apoptosis was suppressed (Fig. 2d). These results suggest that miR-129-2 may function as a tumor suppressor by inhibiting cell growth and promoting cell apoptosis in glioma cells.

### MiR-129-2 inhibits cell migration and invasion in glioma cells in vitro

**Fig. 3:**
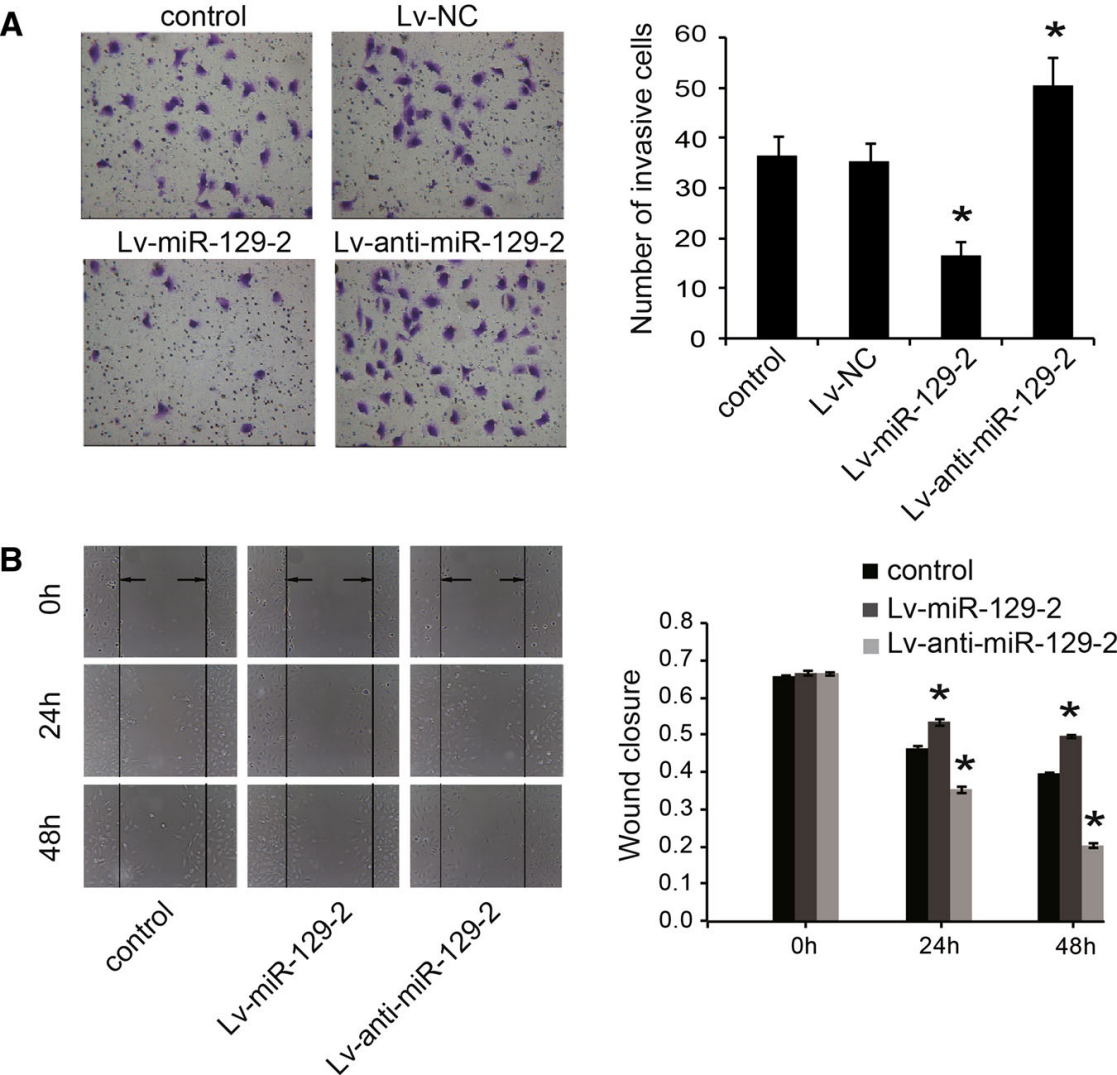
MiR-129-2 inhibits cell migration and invasion in glioma cells. U373 cell migration (**a**) and invasion (**b**) were determined by transwell assays with matrigel and wound-healing assays, respectively. *P < 0.05 versus the control, data shown are mean ± SD. The similar results were also found in U87 cells (data not shown)

We then investigated the effect of miR-129-2 on cell migration and invasion of glioma cells, respectively. Transwell assays with matrigel and wound-healing assays indicated that the enhanced expression of miR-129-2 could significantly inhibit cell invasion and migration compared to control group in U373 and U87 cells (Fig. 3a, b). In contrast, when endogenous miR-129-2 was silenced, the cell invasion (Fig. 3a) and migration (Fig. 3b) were increased. These results suggest that miR-129-2 may function as a tumor suppressor by inhibiting cell migration and invasion in glioma cells.

### MiR-129-2 directly targets HMGB1 and inhibits its expression in glioma cells in vitro

**Fig. 4:**
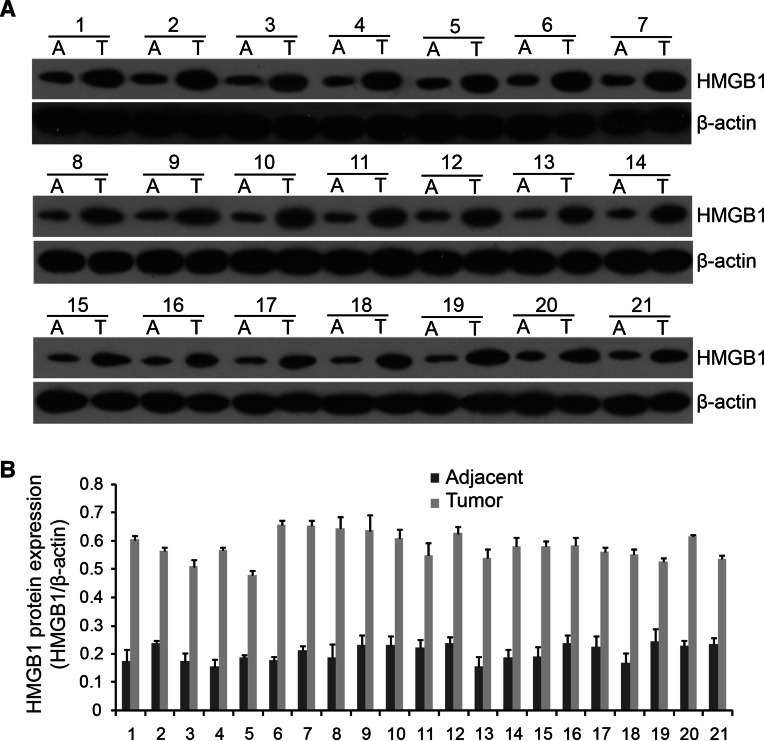
HMGB1 was up-regulated in glioma tumors. **a** Western blot to detect the protein expression levels of HMGB1 in 21 pairs of glioma tumor tissues (T) and their matched adjacent tissues (A). **b** Quantification of the relative expression based on Western blot

**Fig. 5:**
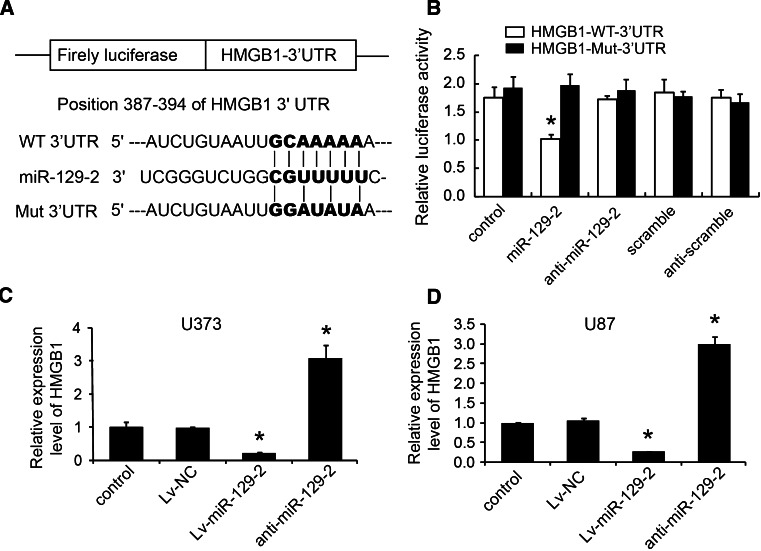
MiR-129-2 directly targets HMGB1 and inhibits its expression in glioma cells. **a** Predicted binding sequences of miR-129-2 in the 3′UTR of HMGB1. Mutation was generated in the seed region (bold bases) of the 3′UTR of HMGB1. **b** The repression of luciferase activity by 3′UTR of HMGB1 was dependent on miR-129-2. Mutated 3′UTR of HMGB1 abrogated miR-129-2-mediated repression of luciferase activity in U373 cells. The similar results were also found in U87 cells (data not shown). **c**, **d** U373 and U87 cells were infected with miR-129-2 or anti-miR-129-2 lentivirus, and the expression levels of miR-129-2 and HMGB1 were analyzed by RTq-PCR. *P < 0.05 versus the control, data shown are mean ± SD

To elucidate the molecular mechanism by which miR-129-2 exerts its inhibitory effect on glioma cells, we predicted potential targets of miR-129-2 and focused on HMGB1 considering the involvement of HMGB1 in the pathogenesis of many human cancers. Besides, Western blot analysis revealed that HMGB1 was significantly up-regulated in glioma tumors compared with those in matched adjacent tissues (Fig. 4). To investigate if miR-129-2 directly targets HMGB1 in glioma cells, we amplified the wild-type HMGB1 3′UTR containing the predicted binding sites of miR-129-2, and its mutant version by the binding site mutagenesis was also constructed (Fig. 5a). They were then cloned downstream to a luciferase reporter, respectively, named HMGB1-WT-3′UTR and HMGB1-Wut-3′UTR vector. These two kinds of vectors were co-transfected with miR-129-2 mimics, miR-129-2 inhibitor (anti-miR-129-2 mimics), or their respective scrambled controls into U373 and U87 cells, respectively. The luciferase activity of miR-129-2 mimic transfected cells was significantly decreased compared with control cells (Fig. 5b). Moreover, miR-129-2-mediated repression of luciferase activity was abolished by the mutant putative binding sites (Fig. 5b). The above results suggest that miR-129-2 directly targets the 3′UTR of HMGB1 in glioma cells.

We then performed RTq-PCR analysis to examine the inhibitory effect of miR-129-2 on endogenous HMGB1 expression in glioma cells. As shown in Fig. 5c, enforced miR-129-2 significantly inhibited HMGB1 expression levels, while reduced miR-129-2 improved HMGB1 levels in U373 and U87 cells. Taken together, our data suggest that miR-129-2 may inhibit HMGB1 expression by directly targeting its 3′UTR in glioma cells.

### Knockdown of HMGB1 inhibits cell migration and invasion of glioma cells in vitro

**Fig. 6:**
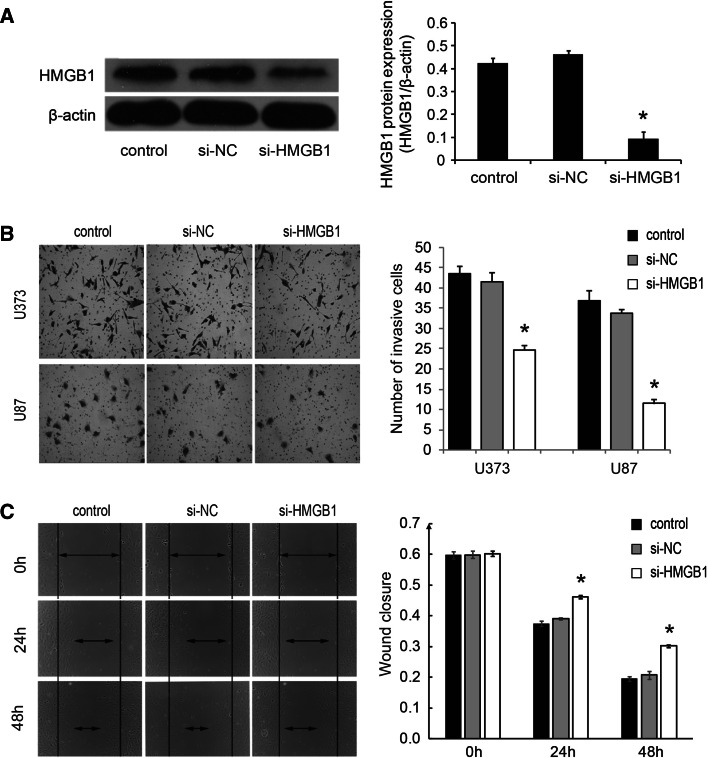
Knockdown of HMGB1 inhibits cell migration and invasion of glioma cells. **a** U373 cells were infected with HMGB1-specific small interfering RNAs (si-HMGB1) and negative control (si-NC), and the expression of HMGB1 was examined by Western blotting. The similar results were also found in U87 cells (data not shown). **b**, **c** The effect of HMGB1 knockdown on the cell migration and invasion was assessed by wound healing and transwell with matrigel assays, respectively. *P < 0.05 versus the control, data shown are mean ± SD

To investigate the functional roles of HMGB1 in glioma cells, we knocked down HMGB1 in U373 and U87 cells by HMGB1-specific small interfering RNAs (si-HMGB1), which was confirmed by Western blot (Fig. 6a). We then performed transwell assays with matrigel (Fig. 6b) and wound-healing assays (Fig. 6c) and found that knockdown of HMGB1 in U373 and U87 cells significantly inhibited cell invasion and migration ability, respectively, which was similar to the effects of miR-129-2 overexpression.

### MiR-129-2 expression is epigenetically regulated by DNA methylation in glioma cells

**Fig. 7:**
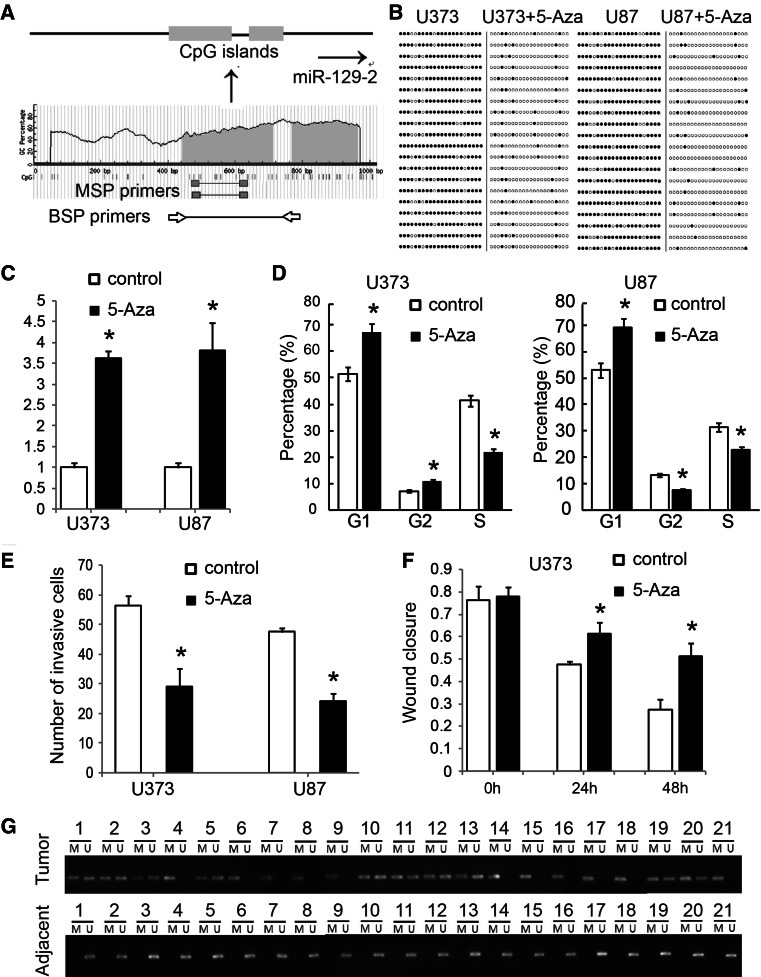
MiR-129-2 expression is epigenetically regulated by DNA methylation in glioma cells. **a** A schematic illustration of CpG islands in the upstream region of miR-129-2 gene and the primers for MSP or BSP. **b** Bisulfite-sequencing PCR detected the methylation status among U373 and U87 cells before and after 5-Aza-dC treatment. Columns indicate CpG dinucleotides 1-22, and horizontal lines represent clones 1-20. Black dots symbolize methylated CpGs, and white dots symbolize unmethylated CpGs. **c** Effect of 5-Aza-dC treatment on miR-129-2 expression in U373 and U87 cells tested by RTq-PCR. **d**, **e**, **f** Effects of 5-Aza-dC treatment on cell cycle, cell migration, and invasion of U373 and U87 cells, **f** for U373, and the similar result was also found in U87 cells (data not shown). **g** MSP results of miR-129-2 methylation in the 21 pairs of glioma tissues (Tumor) and their matched adjacent tissues (Adjacent). M = PCR products amplified by MSP specific for methylated DNA, U = PCR products amplified by MSP specific for unmethylated DNA

The above findings suggested that miR-129-2 is an important regulator in glioma cells. However, the regulatory mechanism of miR-129-2 expression in glioma cells was still unknown. As many miRNAs have been found to be regulated by epigenetic modifications, especially DNA methylation, we then analyzed the regulatory mechanism of miR-129-2 expression from its promoter methylation. We identified two CpG islands in the genomic region spanning the miR-129-2 gene upstream (Fig. 7a). To further investigate whether miR-129-2 was epigenetically regulated in glioma cells, U373 and U87 cells were treated with demethyltransferase inhibitor, 5-aza-2′-deoxycytidine (5-Aza-dC). DNA methylation analysis by bisulfite-cloning and sequencing revealed that the upstream CpG islands of the miR-129-2 gene were demethylated after 5-Aza-dC treatment (Fig. 7b). RTq-PCR analysis revealed that the expression of miR-129-2 was up-regulated in U373 (3.52-fold) and U87 (3.67-fold) cells after the 5-Aza-dC treatment compared with DMSO-treated control group (Fig. 7c). This suggested that epigenetic factors could affect miR-129-2 expression in glioma cells. Functional analysis also revealed that demethylation-induced re-expression of miR-129-2 inhibited cell cycle, cell migration, and invasion of U373 and U87 cells (Fig. 7d–f).

To further examine whether the methylation status of the miR-129-2 upstream region was associated with glioma development, MSP was performed in the 21 pairs of glioma tissues and their matched adjacent tissues. We found that DNA methylation in upstream regions of miR-129-2 frequently occured in 21 tumor tissues (10 partially methylated and 11 completely methylated), while the adjacent tissues were all unmethylated (Fig. 7g).

## Discussion

Glioma, the majority of malignant brain tumors, is one of the most lethal forms of human cancer. Despite of improvements in glioma treatments, the molecular mechanisms underlying its pathology remain poorly understood. In this study, we revealed that miR-129-2 was down-regulated in human glioma cancer, partially due to its DNA promoter hypermethylation. Further, functional studies demonstrated that overexpression of miR-129-2 suppressed cell growth, migration, and invasion and promoted cell apoptosis in the glioma cells, at least partially through targeting the oncogene HMGB1.

In recent years, accumulated evidence revealed that miRNAs play important roles in tumor development and progression. Depending on their mRNA targets, miRNAs can function as tumor suppressors or promoters [21]. MiR-129 family members play an important role in several cancers, including gastric cancer [8, 22], renal cell carcinoma [23], medullary thyroid carcinomas [24], colorectal cancer [25], hepatocellular cancer [26], and breast cancer [27]. In these cancers, miR-129 family members are usually down-regulated and act as a tumor suppressor by decreasing cell growth, inducing apoptosis, or suppressing cell migration and invasion. Consistent with previous reports, our data suggest that miR-129-2 functions as a tumor suppressor in glioma cells by inhibiting cell growth, migration, and invasion and promoting cell apoptosis. Further, prediction and luciferase reporter assay revealed that miR-129-2 could directly target HMGB1 and inhibited its expression.

Recent findings have revealed that HMGB1 dysfunction contributes to cancer initiation and development [28]. HMGB1 overexpression has been observed in many cancers such as colon cancer [29], gastrointestinal stromal tumors [30], melanoma [31], hepatocellular carcinoma [32], and glioma [33], and HMGB1 promotes cell growth and/or migration during the above tumor pathology. In glioma cells, inhibition of HMGB1 was found to suppress the cell growth and migration in vitro [33]. In this study, we inhibited HMGB1 expression by siRNA in U373 and U87 cells and found that knockdown of HMGB1 inhibited cell migration and invasion of glioma cells in vitro, which is consistent with previous report [33]. It has been reported that HMGB1 can induce the growth and migration of cells via its intracellular signaling pathways including NF-κB, MAPK, and ERK [34, 35]. HMGB1 was found to up-regulate the expression of MMP-9, which belongs to matrix metalloproteinases (MMPs) involved in the initiation, invasion, and metastasis of many kinds of cancers, in glioma and gastric cancer cells [33, 36], which may explain its association with invasion and metastasis of glioma tumor. Taken together, the validated involvement of miR-129-1/HMGB1 link in glioma cells may provide potential to use miR-129-2 and HMGB1 as therapeutic targets for glioma.

Epigenetic modifications including DNA hypermethylation are closely associated with gene inactivation. Many reports have revealed that promoter hypermethylation induced down-regulation of tumor suppressive miRNAs closely correlates with carcinogenesis [37–39]. Our data demonstrated that the hypermethylation of the CpG island upstream of miR-129-2 led to the down-regulation of miR-129-2 in glioma patients. Moreover, demethylation of miR-129-2 by 5-Aza-dC treatment increased miR-129-2 expression in glioma cells and resulted in significant inhibitory effects on cell cycle, migration, and invasion. Based on these findings, the methylation status of miR-129-2 may be employed as a potential biomarker in glioma.

In conclusion, our study suggests that miR-129-2 is down-regulated by DNA methylation and functions as a tumor suppressor by targeting HMGB1 in glioma cells. Reintroducing expression of miR-129-2 in glioma cells suppresses cell growth, migration, and invasion and promotes cell apoptosis, which may provide a novel therapeutic strategy for treatment of glioma.
